# Why do people spread false information online? The effects of message and viewer characteristics on self-reported likelihood of sharing social media disinformation

**DOI:** 10.1371/journal.pone.0239666

**Published:** 2020-10-07

**Authors:** Tom Buchanan

**Affiliations:** School of Social Sciences, University of Westminster, London, United Kingdom; Beihang University, CHINA

## Abstract

Individuals who encounter false information on social media may actively spread it further, by sharing or otherwise engaging with it. Much of the spread of disinformation can thus be attributed to human action. Four studies (total *N* = 2,634) explored the effect of message attributes (authoritativeness of source, consensus indicators), viewer characteristics (digital literacy, personality, and demographic variables) and their interaction (consistency between message and recipient beliefs) on self-reported likelihood of spreading examples of disinformation. Participants also reported whether they had shared real-world disinformation in the past. Reported likelihood of sharing was not influenced by authoritativeness of the source of the material, nor indicators of how many other people had previously engaged with it. Participants’ level of digital literacy had little effect on their responses. The people reporting the greatest likelihood of sharing disinformation were those who thought it likely to be true, or who had pre-existing attitudes consistent with it. They were likely to have previous familiarity with the materials. Across the four studies, personality (lower Agreeableness and Conscientiousness, higher Extraversion and Neuroticism) and demographic variables (male gender, lower age and lower education) were weakly and inconsistently associated with self-reported likelihood of sharing. These findings have implications for strategies more or less likely to work in countering disinformation in social media.

## Introduction

Disinformation is currently a critically important problem in social media and beyond. Typically defined as “the deliberate creation and sharing of false and/or manipulated information that is intended to deceive and mislead audiences, either for the purposes of causing harm, or for political, personal or financial gain”, political disinformation has been characterized as a significant threat to democracy [1, p.10]. It forms part of a wider landscape of information operations conducted by governments and other entities [2, 3]. Its intended effects include political influence, increasing group polarisation, reducing trust, and generally undermining civil society [4]. Effects are not limited to online processes. They regularly spill over into other parts of our lives. Experimental work has shown that exposure to disinformation can lead to attitude change [5] and there are many real-world examples of behaviours that have been directly attributed to disinformation, such people as attacking telecommunications masts in response to fake stories about ‘5G causing coronavirus’ [6, 7]. Social media disinformation is very widely used as a tool of influence: computational propaganda has been described as a pervasive and ubiquitous part of modern everyday life [8].

### How does social media disinformation spread?

Once disinformation has initially been seeded online by its creators, one of the ways in which it spreads is through the actions of individual social media users. Ordinary people may propagate the material to their own social networks through deliberate sharing–a core function of platforms such as Facebook and Twitter. Other interactions with it, such as ‘liking’, also trigger the algorithms of social media platforms to display it to other users. This is a phenomenon known as ‘organic reach’ [9]. It can lead to false information spreading exponentially. As an example, analysis of the activity of the Russian ‘Internet Research Agency’ (IRA) disinformation group in the USA between 2015 and 2017 concluded that over 30 million users shared and otherwise interacted with the IRA’s Facebook and Instagram posts, propagating them to their families and friends [4]. There is evidence that false material is spread widely and rapidly through social media due to such human behaviour [10].

### Why do people spread social media disinformation?

When individuals share or interact with disinformation they see online, they have essentially been persuaded to do so by its originators. Influential models of social information processing suggest there are different routes to persuasion [e.g. 11]. Under some circumstances, we may carefully consider the information available. At other times, we make rapid decisions based on heuristics and peripheral cues. When sharing information on social media occurs, it is likely to be spontaneous and rapid, rather than being a considered action that people spend time deliberating over. For example, there are indications of people using the interaction features of Facebook in a relatively unthinking and automatic manner [12]. In such situations, a peripheral route to persuasion is likely be important [13]. Individuals’ choices to share, like and so on will thus be guided primarily by heuristics or contextual cues [14].

Three potentially important heuristics in this context are *consistency*, *consensus* and *authority* [15]. These are not the only heuristics that might possibly influence whether we share false material. However, in each case there is suggestive empirical evidence, and apparent real-world attempts to leverage these phenomena, that make them worth considering.

#### Consistency

Consistency is the extent to which sharing would be consistent with past behaviours or beliefs of the individual. For example, in the USA people with a history of voting Republican might be more likely to endorse and disseminate right-wing messaging [16]. There is a large body of work based on the idea that people prefer to behave in ways consistent with their attitudes [17]. Research has indicated that social media users consider headlines consistent with their pre-existing beliefs as more credible, even when explicitly flagged as being false [18]. In the context of disinformation, this could make it desirable to target audiences sympathetic to the message content.

#### Consensus

Consensus is the extent to which people think their behaviour would be consistent with that of most other people. In the current context, it is possible that seeing a message has already been shared widely might make people more likely to forward it on themselves. In marketing, this influence tactic is known as ‘social proof’ [19]. It is widely used in online commerce in attempts to persuade consumers to purchase goods or services (e.g. by displaying reviews or sales rankings). The feedback mechanisms of social networks can be manipulated to create an illusion of such social support, and this tactic seems to have been used in the aftermath of terror attacks in the UK [20].

Bot networks are used to spread low-credibility information on Twitter through automated means. Bots have been shown to be involved in the rapid spread of information, tweeting and retweeting messages many times [21]. Among humans who see the messages, the high retweet counts achieved through the bot networks might be interpreted as indicating that many other people agree with them. There is evidence which suggests that "each amount of sharing activity by likely bots tends to trigger a disproportionate amount of human engagement" [21, p.4]. Such bot activity could be an attempt to exploit the consensus effect.

It is relatively easy to manipulate the degree of consensus or social proof associated with an online post. Work by the NATO Strategic Communications Centre of Excellence [22] indicated that it was very easy to purchase high levels of false engagement for social media posts (e.g. sharing of posts by networks of fake accounts) and that there was a significant black market for social media manipulation. Thus, if boosting consensus effectively influences organic reach, then it could be a useful tool for both those seeding disinformation and those seeking to spread counter-messages.

#### Authority

Authority is the extent to which the communication appears to come from a credible, trustworthy source [23]. Research participants have been found to report a greater likelihood of propagating a social media message if it came from a trustworthy source [24]. There is evidence of real-world attempts to exploit this effect. In 2018, Twitter identified fraudulent accounts that simulated those of US local newspapers [25], which may be trusted more than national media [26]. These may have been sleeper accounts established specifically for the purpose of building trust prior to later active use.

#### Factors influencing the spread of disinformation

While there are likely to be a number of other variables that also influence the spread of disinformation, there are grounds for believing that consistency, consensus and authority may be important. Constructing or targeting disinformation messages in such a way as to maximise these three characteristics may be a way to increase their organic reach. There is real-world evidence of activity consistent with attempts to exploit them. If these effects do exist, they could also be exploited by initiatives to counter disinformation.

### Who spreads social media disinformation?

Not all individuals who encounter untrue material online spread it further. In fact, the great majority do not. Research linking behavioural and survey data [16] found that less than 10% of participants shared articles from ‘fake news’ domains during the 2016 US presidential election campaign (though of course when extrapolated to the huge user base of social network platforms like Facebook, this is still a very large number of people).

The fact that only a minority of people actually propagate disinformation makes it important to consider what sets them apart from people who don’t spread untrue material further. This will help to inform interventions aimed at countering disinformation. For example, those most likely to be misled by disinformation, or to spread it further, could be targeted with counter-messaging. It is known that the originators of disinformation have already targeted specific demographic groups, in the same way as political campaigns micro-target messaging at those audience segments deemed most likely to be persuadable [27]. For example, it is believed that the ‘Internet Research Agency’ sought to segment Facebook and Instagram users based on race, ethnicity and identity by targeting their messaging to people recorded by the platforms as having certain interests for marketing purposes [4]. They targeted specific communications tailored to those segments (e.g. trying to undermine African Americans’ faith in political processes and suppress their voting in the US presidential election).

#### Digital media literacy

Research has found that older adults, especially those aged over 65, were by far the most likely to spread material originally published by ‘fake news’ domains [16]. A key hypothesis advanced to explain this is that older adults have lower levels of digital media literacy, and are thus less likely to be able to distinguish between true and false information online. While definitions may vary, digital media literacy can be thought of as including “… the ability to interact with textual, sound, image, video and social medias … finding, manipulating and using such information” [28, p. 11] and being a “multidimensional concept that comprised technical, cognitive, motoric, sociological, and emotional aspects” [29, p.834]. Digital media literacy is widely regarded as an important variable mediating the spread and impact of disinformation [e.g. 1]. It is argued that many people lack the sophistication to detect a message as being untruthful, particularly when it appears to come from an authoritative or trusted source. Furthermore, people higher in digital media literacy may be more likely to engage in elaborated, rather than heuristic-driven, processing (cf. work on phishing susceptibility [30]), and thus be less susceptible to biases such as consistency, consensus and authority.

Educating people in digital media literacy is the foundation of many anti-disinformation initiatives. Examples include the ‘News Hero’ Facebook game developed by the NATO Strategic Communications Centre of Excellence (https://www.stratcomcoe.org/news-hero), government initiatives in Croatia and France [8] or the work of numerous fact-checking organisations. The effectiveness of such initiatives relies on two assumptions being met. The first is that lower digital media literacy really does reduce our capacity to identify disinformation. There is currently limited empirical evidence on this point, complicated by the fact that definitions of ‘digital literacy’ are varied and contested, and there are currently no widely accepted measurement tools [28]. The second is that the people sharing disinformation are doing so unwittingly, having been tricked into spreading it. However, it is possible that at least some people *know* the material is untrue, and they spread it anyway. Survey research [31] has found that believing a story was false was not necessarily a barrier to sharing it. People may act like this because they are sympathetic to a story’s intentions or message, or they are explicitly signalling their social identity or allegiance to some political group or movement. If people are deliberately forwarding information that they know is untrue, then raising their digital media literacy would be ineffective as a stratagem to counter disinformation. This makes it important to simultaneously consider users’ beliefs about the veracity of disinformation stories, to inform the design of countermeasures.

#### Personality

It is also known that personality influences how people use social media [e.g. 32]. This makes it possible that personality variables will also influence interactions with disinformation. Indeed, previous research [24] found that people low on Agreeableness reported themselves as more likely to propagate a message. This is an important possibility to consider, because it raises the prospect that individuals could be targeted on the basis of their personality traits with either disinformation or counter-messaging. In a social media context, personality-based targeting of communications is feasible because personality characteristics can be detected from individuals’ social media footprints [33, 34]. Large scale field experiments have shown that personality-targeted advertising on social media can influence user behaviour [35].

The question of which personality traits might be important is an open one. In the current study, personality was approached on an exploratory basis, with no specific hypotheses about effects or their directions. This is because there are a number of different and potentially rival effects that might operate. For example, higher levels of Conscientiousness may be associated with a greater likelihood of posting political material in social media [36] leading to a higher level of political disinformation being shared. However, people higher in Conscientiousness are likely to be more cautious [37] and pay more attention to details [38]. They might therefore also be more likely to check the veracity of the material they share, leading to a lower level of political disinformation being shared.

### Research aims and hypotheses

The overall aim of this project was to establish whether contextual factors in the presentation of disinformation, or characteristics of the people seeing it, make it more likely that they extend its reach. The methodology adopted was scenario-based, with individuals being asked to rate their likelihood of sharing exemplar disinformation messages. A series of four studies was conducted, all using the same methodology. Multiple studies were used to establish whether the same effects were found across different social media platforms (Facebook in Study 1, Twitter in Study 2, Instagram in Study 3) and countries (Facebook with a UK sample in Study 1, Facebook with a US sample in Study 4). Data were also collected on whether participants had shared disinformation in the past. A number of distinct hypotheses were advanced:

H1: Individuals will report themselves as more likely to propagate messages from more authoritative compared to less authoritative sources.

H2: Individuals will report themselves as more likely to propagate messages showing a higher degree of consensus compared to those showing a lower degree of consensus.

H3: Individuals will report themselves as more likely to propagate messages consistent with their pre-existing beliefs compared to inconsistent messages.

H4: Individuals lower in digital literacy will report a higher likelihood of sharing false messages than individuals higher in digital literacy.

Other variables were included in the analysis on an exploratory basis with no specific hypotheses being advanced. In summary, this project asks why ordinary social media users share political disinformation messages they see online. It tests whether specific characteristics of messages or their recipients influence the likelihood of disinformation being further shared online. Understanding any such mechanisms will both increase our understanding of the phenomenon and inform the design of interventions seeking to reduce its impact.

## Study 1

Study 1 tested hypotheses 1–4 with a UK sample, using stimuli relevant to the UK. The study was completed online. Participants were members of research panels sourced through the research company Qualtrics.

### Method

Participants were asked to rate their likelihood of sharing three simulated Facebook posts. The study used an experimental design, manipulating levels of authoritativeness and consensus apparent in the stimuli. All manipulations were between, not within, participants. Consistency with pre-existing beliefs was not manipulated. Instead, the political orientation of the stimuli was held constant, and participants’ scores on conservative political orientation were used as an index of consistency between messages and participant beliefs. The effects of these variables on self-rated likelihood of sharing the stimuli, along with those of a number of other predictors, were assessed using multiple regression. The primary goal of the analysis was to identify variables that statistically significantly explained variance in the likelihood of sharing disinformation. The planned analysis was followed by supplementary and exploratory analyses. All analyses were conducted using SPSS v.25 for Mac. For all studies reported in this paper, ethical approval came from both the University of Westminster Research Ethics Committee (ETH1819-1420) and the Lancaster University Security Research Ethics Committee (BUCHANAN 2019 07 23). Consent was obtained, via an electronic form, from anonymous participants.

#### Materials

A short questionnaire was used to capture demographic information (gender; country of residence; education; age; occupational status; political orientation expressed as right, left or centre; frequency of Facebook use). Individual differences in personality, political orientation, and digital / new media literacy were measured using established validated questionnaires. Ecologically valid stimuli were used, with their presentation being modified across conditions to vary authoritativeness and consensus markers.

Personality was measured using a 41-item Five-Factor personality questionnaire [38] derived from the International Personality Item Pool [37]. The measure provides indices of Extraversion, Neuroticism, Openness to Experience, Agreeableness and Conscientiousness that correlate well with the domains of Costa and McCrae's [39] Five Factor Model.

Conservatism was measured using the 12-item Social and Economic Conservatism Scale (SECS) [40], which is designed to measure political orientation along a left-right; liberal-conservative continuum. It was developed and validated using a US sample. In pilot work for the current study, mean scores for individuals who reported voting for the Labour and Conservative parties in the 2017 UK general election were found to differ in the expected manner (*t*_(28)_ = -2.277, *p* = .031, *d* = 0.834). This provides evidence of its appropriateness for use in UK samples. While the measure provides indices of different aspects of conservatism, it also provides an overall conservatism score which was used in this study.

Digital media literacy was measured using the 35-item New Media Literacy Scale (NMLS) [29]. This is a theory-based self-report measure of competences in using, critically interrogating, and creating digital media technologies and messaging. In pilot work with a UK sample, it was found to distinguish between individuals high or low in social media (Twitter) use, providing evidence of validity (*t*_(194)_ = -3.847, *p* < .001, *d* = .55). While the measure provides indices of different aspects of new media literacy, it also provides an overall score which was used in this study.

Participants were asked to rate their likelihood of sharing three genuine examples of ‘fake news’ that had been previously published online. An overall score for their likelihood of sharing the stimuli was obtained by summing the three ratings, creating a combined score. This was done, and a set of three stimuli was used, to reduce the likelihood that any effects found were peculiar to a specific story. The stimuli were sourced from the website Infowars.com (which in some cases had republished them from other sources). Infowars.com has been described [41] as a high-exposure site strongly associated with the distribution of ‘fake news’. Rather than full articles, excerpts (screenshots) were used that had the size and general appearance of what respondents might expect to see on social media sites. The excerpts were edited to remove any indicators of the source, metrics such as the numbers of shares, date, and author. All had a right-wing orientation (so that participant conservatism could be used as a proxy for consistency between the messages and existing beliefs). This was established in pilot work rating their political orientation and likelihood of being shared. The three stories were among seven rated by a UK sample (*N* = 30) on an 11-point scale asking “To what extent do you think this post was designed to appeal to people with right wing (politically conservative) views?” anchored at “Very left wing oriented” and “Very right wing oriented”. All seven were rated statistically significantly above the politically-neutral midpoint of the scale. Of the three stimuli selected for use in this study, a one-sample *t-*test showed that the least right-wing was statistically significantly higher than the midpoint, (*t*_(39)_ = 4.385, *p* < .001, *d* = 0.70).

One of the stimuli was a picture of masked and hooded men titled “Censored video: watch Muslims attack men, women & children in England”. One was a picture of many people walking down a road, titled “Revealed: UN plan to flood America with 600 million migrants”, with accompanying text describing a plan to “flood America and Europe with hundreds of millions of migrants to maintain population levels”. The third was a picture of the Swedish flag titled “‘Child refugee’ with flagship Samsung phone and gold watch complains about Swedish benefits rules”, allegedly describing a 19 year-old refugee’s complaints.

The authoritativeness manipulation was achieved by pairing the stimuli with sources regarded as relatively high or low in authoritativeness. The source was shown above the stimulus being rated, in the same way as the avatar and username of someone who had posted a message would be on Facebook. The lower authoritativeness group were slight variants on real usernames that had previously retweeted either stories from Infowars.com or another story known to be untrue. The original avatars were used. The exemplars used in this study were named ‘Tigre’ (with an avatar of an indistinct picture of a female face), ‘jelly beans’ (a picture of some jelly beans) and ‘ChuckE’ (an indistinct picture of a male face). The higher authoritativeness group comprised actual fake accounts set up by the Internet Research Agency (IRA) group to resemble local news sources, selected from a list of suspended IRA accounts released by Twitter. The exemplars used in this study were ‘Los Angeles Daily’, ‘Chicago Daily News’ and ‘El Paso Top News’. Pilot work was conducted with a sample of UK participants (*N* = 30) who each rated a selection of 9 usernames, including these 6, for the extent to which each was “likely to be an authoritative source—that is, likely to be a credible and reliable source of information”. A within-subjects *t*-test indicated that mean authoritativeness ratings for the ‘higher’ group were statistically significantly higher than the ‘lower’ group (*t*_(29)_ = -11.181, *p <* .001, *d*_*z*_ = 2.04).

The consensus manipulation was achieved by pairing the stimuli with indicators of the number of shares and likes the story had. The indicators were shown below the stimulus being rated, in the same way as they normally would be on Facebook. In the low consensus conditions, low numbers of likes (1, 3, 2) and shares (2, 0, 2) were displayed. In the high consensus conditions, higher (but not unrealistic) numbers of likes (104K, 110K, 63K) and shares (65K, 78K, 95K) were displayed. The information was presented using the same graphical indicators as would be the case on Facebook, accompanied by the (inactive) icons for interacting with the post, in order to maximise ecological validity.

#### Procedure

The study was conducted completely online, using materials hosted on the Qualtrics research platform. Participants initially saw an information page about the study, and on indicating their consent proceeded to the demographic items. They then completed the personality, conservatism and new media literacy scales. Each of these was presented on a separate page, except the NMLS which was split across three pages.

Participants were then asked to rate the three disinformation items. Participants were randomized to different combinations of source and story within their assigned condition. For example, Participant A might have seen Story 1 attributed to Source 1, Story 2 attributed to Source 2, and Story 3 attributed to Source 3; while Participant B saw Story 1 attributed to Source 2, Story 2 attributed to Source 1, and Story 3 attributed to Source 3. Each participant saw the same three stories paired with one combination of authoritativeness and consensus. There were 24 distinct sets of stimuli.

Each participant saw an introductory paragraph stating “A friend of yours recently shared this on Facebook, commenting that they thought it was important and asking all their friends to share it:”. Below this was the combination of source, story, and consensus indicators, presented together in the same way as a genuine Facebook post would be. They then rated the likelihood of them sharing the post to their own public timeline, on an 11-point scale anchored at ‘Very Unlikely’ and ‘Very Likely’. This was repeated for the second and third stimuli, each on a separate page. Having rated each one, participants were then shown all three stimuli again, this time on the same page. They were asked to rate each one for “how likely do you think it is that the message is accurate and truthful” and “how likely do you think it is that you have seen it before today”, on 5-point scales anchored at ‘Not at all likely’ and ‘Very likely’.

After rating the stimuli, participants were asked two further questions: “Have you ever shared a political news story online that you later found out was made up?”, and “And have you ever shared a political news story online that you thought AT THE TIME was made up?”, with ‘yes’ or ‘no’ response options. This question format directly replicated that used in Pew Research Centre surveys dealing with disinformation [e.g. 31].

Finally, participants were given the opportunity once again to give or withdraw their consent for participation. They then proceeded to a debriefing page. It was only at the debriefing stage that they were told the stories they had seen were untrue: no information about whether the stimuli were true or false had been presented prior to that point.

#### Data screening and processing

Prior to delivery of the sample, Qualtrics performed a series of quality checks and ‘data scrubbing’ procedures to remove and replace participants with response patterns suggesting inauthentic or inattentive responding. These included speeding checks and examination of response patterns. On delivery of the initial sample (*N* = 688) further screening procedures were performed. Sixteen respondents were identified who had responded with the same scores to substantive sections of the questionnaire (‘straightlining’). These were removed, leaving *N* = 672. These checks and exclusions were carried out prior to any data analysis. Where participants had missing data on any variables, they were omitted only from analyses including those variables. Thus, *N*s vary slightly throughout the analyses.

#### Participants

The target sample size was planned to exceed N = 614, which would give 95% power to detect *R*^*2*^ = .04 (a benchmark for the minimum effect size likely to have real-world importance in social science research [42]), in the planned multiple regression analysis with 11 predictors. Qualtrics was contracted to provide a sample of Facebook users that was broadly representative of the UK 2011 census population in terms of gender; the split between those who had post-secondary-school education and those who had not; and age profile (18+). Quotas were used to assemble a sample comprising approximately one third each self-describing as left-wing, centre and right-wing in their political orientation. Participant demographics are shown in Table 1, column 1.

**Table 1:** Demographic data, Studies 1–4.

	Study 1	Study 2	Study 3	Study 4
	(UK, *N* = 672)	(UK, *N* = 674)	(UK, *N* = 650)	(US, *N* = 638)
**Gender**				
Male	317 (47.2%)	312 (46.3%)	225 (34.6%)	283 (44.4%)
Female	353 (52.5%)	359 (53.3%)	422 (64.9%)	354 (55.5%)
Other	1 (0.1%)	2 (0.3%)	3 (0.5%)	1 (0.2%)
Prefer not to say	1 (0.1%)	1 (0.1%)	0 (0%)	0 (0%)
**Age group**				
18–29	138 (20.5%)	184 (27.3%)	291 (44.8%)	143 (22.4%)
30–39	127 (18.9%)	173 (25.7%)	212 (32.6%)	120 (18.8%)
40–49	129 (19.2%)	176 (26.1%)	81 (12.5%)	106 (16.6%)
50–59	130 (19.3%)	99 (14.7%)	49 (7.5%)	96 (15.0%)
60–69	96 (14.3%)	37 (5.5%)	15 (2.3%)	160 (25.1%)
70+	49 (7.3%)	5 (0.7%)	1 (0.2%)	12 (1.9%)
Unanswered	3 (0.4%)	0 (0%)	1 (0.2%)	1 (0.2%)
**Facebook use**				
Every few weeks	21 (3.1%)	-	-	21 (3.3%)
A few times a week	77 (11.5%)	-	-	52 (8.2%)
About once a day	124 (18.5%)	-	-	128 (20.1%)
Several times a day	450 (67.0%)	-	-	437 (68.5%)
**Twitter use**				
Every few weeks		53 (7.9%)	-	-
A few times a week		165 (24.5%)	-	-
About once a day		151 (22.4%)	-	-
Several times a day		305 (45.3%)	-	-
**Instagram use**				
Every few weeks	-	-	22 (3.4%)	-
A few times a week	-	-	84 (12.9%)	-
About once a day	-	-	145 (22.3%)	-
Several times a day	-	-	399 (61.4%)	-
**Political ideology**				
Left	228 (33.9%)	247 (36.6%)	228 (35.1%)	199 (31.2%)
Centre	227 (33.8%)	237 (35.2%)	255 (39.2%)	229 (35.9%)
Right	216 (32.1%)	190 (28.2%)	167 (25.7%)	207 (32.4%)
Unanswered	1 (0.2%)	0 (0%)	0 (0%)	3 (0.5%)
**Highest education**				
Less than high school	11 (1.6%)	1 (0.1%)	2 (0.3%)	12 (1.9%)
High school / secondary school	394 (58.6%)	113 (16.8%)	119 (18.3%)	228 (35.7%)
Some college or University	75 (11.2%)	158 (23.4%)	167 (25.7%)	144 (22.6%)
College or University degree	142 (21.1%)	282 (41.8%)	262 (40.3%)	179 (28.1%)
Master’s degree	38 (5.7%)	93 (13.8%)	82 (12.6%)	45 (7.1%)
Doctoral degree	6 (0.9%)	16 (2.3%)	8 (1.2%)	11 (1.7%)
Professional degree (JD, MD)	5 (0.7%)	11 (1.6%)	10 (1.5%)	19 (3.0%)
Unanswered	1 (0.1%)	0 (0%)	0 (0%)	0 (0%)
**Occupation**				
Employed for Wages	368 (54.8%)	430 (63.8%)	379 (58.3%)	312 (48.9%)
Self-employed	39 (5.8%)	77 (11.4%)	64 (9.8%)	55 (8.6%)
Unemployed but looking for work	33 (4.9%)	28 (4.2%)	27 (4.2%)	36 (5.6%)
Home-maker	70 (10.4%)	44 (6.5%)	39 (6.0%)	45 (7.1%)
Student	26 (3.9%)	60 (8.9%)	113 (17.4%)	23 (3.6%)
Retired	96 (14.3%)	23 (3.4%)	10 (1.5%)	121 (19%)
Unable to work for health or other reasons	39 (5.8%)	12 (1.8%)	18 (2.8%)	46 (7.2%)
Unanswered	1 (0.1%)	0 (0%)	0 (0%)	0 (0%)

### Results

Descriptive statistics for participant characteristics (personality, conservatism, new media literacy and age) and their reactions to the stimuli (likelihood of sharing, belief the stories were likely to be true, and rating of likelihood that they had seen them before) are summarised in Table 2. All scales had acceptable reliability. The main dependent variable, likelihood of sharing, had a very skewed distribution with a strong floor effect: 39.4% of the participants indicated they were ‘very unlikely’ to share any of the three stories they saw. This is consistent with findings on real-world sharing that indicate only a small proportion of social media users will actually share disinformation [e.g. 16], though it gives a dependent variable with less than ideal distributional properties.

**Table 2:** Descriptive statistics for participant characteristics and response variables, Study 1.

					Range			
	*N*	*M*	*SD*	*α*	Potential	Actual	Skew	Kurtosis
Openness	668	22.55	5.22	0.70	7–35	8–35	-0.20	-0.16
Conscientiousness	667	36.03	6.10	0.79	10–50	12–50	-0.14	-0.04
Extraversion	669	26.56	6.33	0.81	9–45	9–44	-0.19	0.10
Agreeableness	669	26.85	4.49	0.78	7–35	13–35	-0.52	-0.16
Neuroticism	669	21.97	7.02	0.87	8–40	8–40	0.14	-0.39
Conservatism	670	651.00	146.26	0.72	0–1200	160–1090	-0.43	0.12
New Media Literacy	652	124.76	20.18	0.95	35–175	55–172	-0.26	0.01
Likelihood of sharing	672	8.92	7.49		3–33	3–33	1.32	0.97
Belief stories true	672	6.82	3.18		3–15	3–15	0.60	-0.40
Likelihood seen before	672	6.10	3.34		3–15	3–15	0.83	-0.40
Age	669	45.32	15.91			18–86	0.13	-0.94

To simultaneously test hypotheses 1–4 a multiple regression analysis was carried out. This evaluated the extent to which digital media literacy (NMLS), authority of the message source, consensus, belief in veracity of the messages, consistency with participant beliefs (operationalised as the total SECS conservatism scale score), age and personality (Extraversion, Conscientiousness, Agreeableness, Openness to Experience and Neuroticism), predicted self-rated likelihood of sharing the posts. This analysis is summarised in Table 3. Checks were performed on whether the dataset met the assumptions required by the analysis (absence of collinearity, independence of residuals, heteroscedasticity and non-normal distribution of residuals). Despite the skewed distribution of the dependent variable, no significant issues were detected.

**Table 3:** Predictors of rated likelihood of sharing stimuli, Study 1.

	*B*	*SE*		*β*	*t*	*p*
Constant	1.596	3.363			0.475	.635
New Media Literacy	0.026	0.013		0.069	1.908	.057
Conservatism	0.005	0.002		0.090	2.505	.013
Openness	-0.094	0.053		-0.066	-1.786	.075
Neuroticism	-0.037	0.042		-0.035	-0.885	.377
Extraversion	0.076	0.044		0.064	1.744	.082
Conscientiousness	-0.064	0.047		-0.051	-1.362	.174
Agreeableness	-0.183	0.062		-0.108	-2.947	.003
Authoritativeness	0.091	0.478		0.006	0.189	.850
Consensus	0.606	0.480		0.040	1.263	.207
Belief stories true	1.292	0.080		0.543	16.139	.000
Age	0.002	0.017		0.003	0.090	.928
*R*^*2*^			.377			
Adjusted *R*^*2*^			.366			
*F*			34.353			.000

Authoritativeness and Consensus conditions coded as dummy variables; higher = 1, lower = 0 in each case. *N* = 636.

However, exploratory analyses indicated that inclusion of other variables in the regression model might be warranted. It is well established that there are gender differences on a number of personality variables. Furthermore, in the current sample men and women differed in their level of conservatism (*M =* 669.10, *SD =* 150.68 and *M =* 636.50, *SD =* 138.31 respectively; *t*_(666)_ = 2.914, *p* = .004), their self-rated likelihood of sharing (*M =* 10.41, *SD =* 8.33 and *M =* 7.60, *SD =* 6.38 respectively; *t*_(589.60)_ = 4.928, *p* < .001; adjusted *df* used due to heterogeneity of variance, Levene’s *F* = 35.99, *p* < .001), and their belief that the stories were true (*M =* 7.16, *SD =* 3.22 and *M =* 6.52, *SD =* 3.12 respectively; *t*_(668)_ = 2.574, *p* = .010). Education level was found to correlate positively with NMLS scores (*r =* .210, *N* = 651, *p* < .001). Level of Facebook use correlated significantly with age (*r =* -.126, *N = 669*, *p* = .001), education (*r* = .082, *N* = 671, *p* = .034), NMLS (*r* = .170, *N* = 652, *p* < .001), with likelihood of sharing (*r* = .079, *N* = 672, *p* = .040), and with likelihood of having seen the stimuli before (*r* = .107, *N* = 672, *p* = .006). Self-reported belief that respondents had seen the stories before also correlated significantly with likelihood of sharing (*r* = .420, *N* = 672, *p* < .001), and a number of other predictor variables.

Accordingly, a further regression analysis was performed, including these additional predictors (gender, education, level of Facebook use, belief they had seen the stories before). Given inclusion of gender as a predictor variable, the two respondents who did not report their gender as either male or female were excluded from further analysis. The analysis, summarised in Table 4, indicated that the model explained 43% of the variance in self-reported likelihood of sharing the three disinformation items. Neither the authoritativeness of the story source, nor consensus information associated with the stories, was a significant predictor.

**Table 4:** Predictors of rated likelihood of sharing stimuli (extended predictor set), Study 1.

	*B*	*SE*		*β*	*t*	*p*
Constant	0.395	3.473			0.114	.909
New Media Literacy	0.012	.013		.031	0.861	.390
Conservatism	0.005	.002		.097	2.828	.005
Openness	-0.059	.052		-.041	-1.136	.257
Neuroticism	-0.007	.041		-.007	-0.180	.857
Extraversion	0.075	.042		.064	1.796	.073
Conscientiousness	-0.017	.045		-.014	-0.376	.707
Agreeableness	-0.123	.061		-.072	-2.023	.044
Authoritativeness	0.319	.463		.021	0.690	.490
Consensus	0.563	.462		.037	1.219	.223
Belief stories true	1.059	.084		.444	12.573	.000
Age	-0.014	.017		-.029	-0.778	.437
Gender (M = 1, F = 2)	-1.602	.508		-.106	-3.153	.002
Education	-0.709	.224		-.103	-3.160	.002
Facebook use	0.369	.294		.041	1.257	.209
Likelihood seen before	0.479	.079		.211	6.053	.000
*R*^*2*^			.431			
Adjusted *R*^*2*^			.418			
*F*			31.258			.000

Authoritativeness and Consensus conditions coded as dummy variables; higher = 1, lower = 0 in each case. *N =* 634.

Consistency of the items with participant attitudes (conservatism) was important, with a positive and statistically significant relationship between conservatism and likelihood of sharing. The only personality variable predicting sharing was Agreeableness, with less agreeable people giving higher ratings of likelihood of sharing. In terms of demographic characteristics, gender and education were statistically significant predictors, with men and less-educated people reporting a higher likelihood of sharing. Finally, people reported a greater likelihood of sharing the items if they believed they were likely to be true, and if they thought they had seen them before.

Participants had also been asked about their historical sharing of untrue political stories, both unknowing and deliberate. 102 out of 672 participants (15.2%) indicated that they had ever ‘shared a political news story online that they later found out was made up’, while 64 out of 672 indicated they had shared one that they ‘thought AT THE TIME was made up’ (9.5%). Predictors of whether or not people had shared untrue material under both sets of circumstances were examined using logistic regressions, with the same sets of participant-level predictors.

Having unknowingly shared untrue material (Table 5) was significantly predicted by lower Conscientiousness, lower Agreeableness, and lower age. Having shared material known to be untrue at the time (Table 6) was significantly predicted by lower Agreeableness and lower age.

**Table 5:** Binary logistic regression: Predictors of whether participants had previously shared political stories they subsequently discovered were false, Study 1.

	*B*	*S*.*E*.	*Wald*	*df*	*p*	*Exp(B)*	*95% C*.*I*.*for EXP(B)*
New Media Literacy	0.004	0.007	0.415	1	.519	1.004	[0.991, 1.018]
Conservatism	0.001	0.001	0.339	1	.560	1.001	[0.999, 1.002]
Openness	-0.040	0.027	2.157	1	.142	0.961	[0.912, 1.013]
Neuroticism	-0.007	0.023	0.088	1	.767	0.993	[0.950, 1.039]
Extraversion	0.036	0.023	2.336	1	.126	1.036	[0.990, 1.085]
Conscientiousness	-0.053	0.024	4.971	1	.026	0.949	[0.906, 0.994]
Agreeableness	-0.085	0.030	8.26	1	.004	0.918	[0.866, 0.973]
Age	-0.024	0.009	7.416	1	.006	0.976	[0.960, 0.993]
Gender (M = 1, F = 2)	-0.463	0.258	3.213	1	.073	0.629	[0.379, 1.044]
Education	0.082	0.108	0.582	1	.446	1.086	[0.879, 1.341]
Facebook use	0.336	0.176	3.644	1	.056	1.400	[0.991, 1.978]
Constant	1.463	1.736	0.71	1	.399	4.320	

*N =* 634.

**Table 6:** Binary logistic regression: Predictors of whether participants had previously shared political stories they knew at the time were false, Study 1.

	*B*	*S*.*E*.	*Wald*	*df*	*p*	*Exp(B)*	*95% C*.*I*.*for EXP(B)*
New Media Literacy	0.003	0.008	0.138	1	.711	1.003	[0.987, 1.019]
Conservatism	0.000	0.001	0.001	1	.981	1.000	[0.998, 1.002]
Openness	0.013	0.037	0.117	1	.732	1.013	[0.942, 1.088]
Neuroticism	-0.001	0.030	0.002	1	.965	0.999	[0.942, 1.059]
Extraversion	0.054	0.032	2.854	1	.091	1.056	[0.991, 1.124]
Conscientiousness	-0.043	0.031	1.983	1	.159	0.958	[0.902, 1.017]
Agreeableness	-0.173	0.039	20.169	1	.000	0.841	[0.780, 0.907]
Age	-0.042	0.012	12.827	1	.000	0.959	[0.938, 0.981]
Gender (M = 1, F = 2)	-0.602	0.325	3.420	1	.064	0.548	[0.290, 1.037]
Education	0.123	0.128	0.918	1	.338	1.131	[0.879, 1.455]
Facebook use	0.181	0.214	0.715	1	.398	1.198	[0.788, 1.822]
Constant	2.920	2.179	1.796	1	.180	18.548	

*N =* 634.

### Discussion

The main analysis in this study (Table 4) provided limited support for the hypotheses. Contrary to hypotheses 1, 2, and 4, neither consensus markers, authoritativeness of source, nor new media literacy were associated with self-rated likelihood of sharing the disinformation stories. However, in line with hypothesis 3, higher levels of conservatism were associated with higher likelihood of sharing disinformation. This finding supports the proposition that we are more likely to share things that are consistent with our pre-existing beliefs, as all the stimuli were right-wing in orientation. An alternative explanation might be that more conservative people are simply more likely to share disinformation. However, as well as lacking a solid rationale, this explanation is not supported by the fact that conservatism did not seem to be associated with self-reported historical sharing (Tables 5 and 6).

The strongest predictors of likelihood of sharing were belief that the stories were true, and likelihood of having seen them before. Belief in the truth of the stories provides further evidence for the role of consistency (hypothesis 3), in that we are more likely to share things we believe are true. The association with likely previous exposure to the materials is consistent with other recent research [43, 44] that found that prior exposure to ‘fake news’ headlines led to higher belief in their accuracy and reduced belief that it would be unethical to share them.

Of the personality variables, only Agreeableness was a significant predictor, with less agreeable people rating themselves are more likely to share the stimuli. This is consistent with previous findings [24] that less agreeable people reported they were more likely to share a critical political message.

Lower education levels were associated with a higher self-reported likelihood of sharing. It is possible that less educated people may be more susceptible to online influence, given work finding that less educated people were more influenced by micro-targeted political advertising on Facebook [45].

Finally, gender was found to be an important variable, with men reporting a higher likelihood of sharing the disinformation messages than women. This was unanticipated: while there are a number of gender-related characteristics (e.g. personality traits) that were thought might be important, there were no *a priori* grounds to expect that gender itself would be a predictor variable.

Study 1 also examined predictors of reported historical sharing of false political information. Consistent with real-world data [16], and past representative surveys [e.g. 31], a minority of respondents reported such past sharing. Unknowingly sharing false political stories was predicted by low Conscientiousness, low Agreeableness, and lower age, while knowingly sharing false material was predicted only by lower Agreeableness and lower age. The effect of Agreeableness is consistent with the findings from the main analysis and from [24]. The finding that Conscientiousness influenced accidental, but not deliberate, sharing is consistent with the idea that less conscientious people are less likely to check the details or veracity of a story before sharing it. Clearly this tendency would not apply to deliberate sharing of falsehoods. The age effect is harder to explain, especially given evidence [16] that older people were more likely to share material from fake news sites. One possible explanation is that younger people are more active on social media, so would be more likely to share any kind of article. Another possibility is that they are more likely to engage in sharing humorous political memes, which could often be classed as false political stories.

## Study 2

Study 2 set out to repeat Study 1, but presented the materials as if they had been posted on Twitter rather than Facebook. The purpose of this was to test whether the observed effects applied across different platforms. Research participants have reported using ‘likes’ on Twitter in a more considered manner than on Facebook [12], raising the possibility that heuristics might be less important for this platform. The study was completed online, using paid respondents sourced from the Prolific research panel (www.prolific.co).

### Method

The methodology exactly replicated that of Study 1, except in the case of details noted below. The planned analysis was revised to include the expanded set of predictors eventually used in Study 1 (see Table 4).

#### Materials

Measures and materials were the same as used in Study 1. The key difference from Study 1 was in the presentation of the three stimuli, which were portrayed as having been posted to Twitter rather than Facebook. For the authoritativeness manipulation, the screen names of the sources were accompanied by @usernames, as is conventional on Twitter. For the consensus manipulation, ‘retweets’ were displayed rather than ‘shares’, and the appropriate icons for Twitter were used. Participants also indicated their level of Twitter, rather than Facebook, use.

#### Procedure

The procedure replicated Study 1, save that in this case the NMLS was presented on a single page. Before participants saw each of the three disinformation items, the introductory paragraph stated “A friend of yours recently shared this on Twitter, commenting that they thought it was important and asking all their friends to retweet it:”, and they were asked to indicate the likelihood of them ‘retweeting’ rather than ‘sharing’ the post.

#### Data screening and processing

Data submissions were initially obtained from 709 participants. A series of checks were performed to ensure data quality, resulting in a number of responses being excluded. One individual declined consent. Eleven were judged to have responded inauthentically, with the same responses to all items in substantive sections of the questionnaire (‘straightlining’). Twenty were not active Twitter users: three individuals visited Twitter ‘not at all’ and seventeen ‘less often’ than every few weeks. Three participants responded unrealistically quickly, with response durations shorter than four minutes (the same value used as a speeding check by Qualtrics in Study 1). All of these respondents were removed, leaving *N* = 674. These checks and exclusions were carried out prior to any data analysis.

#### Participants

The target sample size was planned to exceed *N* = 614, as in Study 1. No attempt was made to recruit a demographically representative sample: instead, sampling quotas were used to ensure the sample was not homogenous with respect to education (pre-degree vs. undergraduate degree or above), age (under 40 vs. over 40) and political preference (left, centre or right wing orientation). Additionally, participants had to be UK nationals resident in the UK; active Twitter users; and not participants in prior studies related to this one. Each participant received a reward of £1.25. Participant demographics are shown in Table 1 (column 2). For the focal analysis in this study, the sample size conferred 94.6% power to detect *R*^*2*^ = .04 in a multiple regression with 15 predictors (2-tailed, alpha = .05).

### Results

Descriptive statistics are summarised in Table 7. All scales had acceptable reliability. The main dependent variable, likelihood of sharing, again had a very skewed distribution with a strong floor effect.

**Table 7:** Descriptive statistics for participant characteristics and response variables, Study 2.

					Range			
	*N*	*M*	*SD*	*α*	Potential	Actual	Skew	Kurtosis
Openness	674	24.42	5.68	.76	7–35	9–35	-0.20	-0.54
Conscientiousness	674	35.85	6.42	.84	10–50	12–50	-0.35	0.06
Extraversion	674	26.24	7.05	.88	9–45	9–45	-0.01	-0.42
Agreeableness	674	27.33	3.94	.76	7–35	13–35	-0.51	0.19
Neuroticism	674	21.66	7.00	.88	8–40	8–40	0.13	-0.68
Conservatism	674	612.72	173.30	.82	0–1200	140–1150	-0.11	-0.21
New Media Literacy	674	134.55	17.05	.93	35–175	59–173	-0.14	0.09
Likelihood of retweeting	674	6.61	5.94		3–33	3–33	1.98	3.54
Belief stories true	674	5.87	2.68		3–15	3–15	0.86	0.21
Likelihood seen before	674	5.70	3.00		3–15	3–15	1.06	0.43
Age	674	38.85	12.64			18–75	0.37	-0.59

To simultaneously test hypotheses 1–4, a multiple regression analysis was carried out using the expanded predictor set from Study 1. Given inclusion of gender as a predictor variable, the three respondents who did not report their gender as either male or female were excluded from further analysis. The analysis, summarised in Table 8, indicated that the model explained 46% of the variance in self-reported likelihood of sharing the three disinformation items. Neither the authoritativeness of the story source, nor consensus information associated with the stories, nor new media literacy, was a significant predictor. Consistency of the items with participant attitudes (conservatism) was important, with a positive and statistically significant relationship between conservatism and likelihood of sharing. No personality variable predicted ratings of likelihood of sharing. In terms of demographic characteristics, gender and education were statistically significant predictors, with men and less-educated people reporting a higher likelihood of sharing. Finally, people reported a greater likelihood of sharing the items if they believed they were likely to be true, and if they thought they had seen them before.

**Table 8:** Predictors of rated likelihood of retweeting stimuli (extended predictor set), Study 2.

	*B*	*SE*		*β*	*t*	*p*
Constant	-3.027	2.717			-1.114	.266
New Media Literacy	0.005	0.011		0.013	0.421	.674
Conservatism	0.004	0.001		0.107	2.788	.005
Openness	0.021	0.037		0.020	0.581	.561
Neuroticism	0.013	0.030		0.015	0.441	.659
Extraversion	0.018	0.027		0.021	0.663	.508
Conscientiousness	0.038	0.031		0.042	1.231	.219
Agreeableness	-0.070	0.048		-0.047	-1.449	.148
Authoritativeness	-0.402	0.345		-0.034	-1.165	.244
Consensus	0.354	0.343		0.030	1.032	.302
Belief stories true	1.238	0.074		0.557	16.742	.000
Age	0.015	0.015		0.032	1.031	.303
Gender (M = 1, F = 2)	-1.026	0.368		-0.086	-2.789	.005
Education	-0.375	0.162		-0.069	-2.316	.021
Twitter use	0.017	0.177		0.003	0.095	.925
Likelihood seen before	0.191	0.059		0.096	3.226	.001
*R*^*2*^			.459			
Adjusted *R*^*2*^			.447			
*F*			37.070			.000

Authoritativeness and Consensus conditions coded as dummy variables; higher = 1, lower = 0 in each case. *N* = 671.

Participants had also been asked about their historical sharing of untrue political stories, both unknowing and deliberate. 102 out of 674 participants (15.1%) indicated that they had out ever ‘shared a political news story online that they later found out was made up’, while 42 out of 674 indicated they had shared one that they ‘thought AT THE TIME was made up’ (6.2%). Predictors of whether or not people had shared untrue material under both sets of circumstances were examined using logistic regressions, with the same sets of participant-level predictors.

Having unknowingly shared untrue material (Table 9) was significantly predicted by higher Extraversion and higher levels of Twitter use. Having shared material known to be untrue at the time (Table 10) was significantly predicted by higher Neuroticism and being male.

**Table 9:** Binary logistic regression: Predictors of whether participants had previously shared political stories they subsequently discovered were false, Study 2.

	*B*	*S*.*E*.	*Wald*	*df*	*p*	*Exp(B)*	*95% C*.*I*.*for EXP(B)*
New Media Literacy	0.002	0.007	0.055	1	.815	1.002	[0.988, 1.016]
Conservatism	0.001	0.001	3.038	1	.081	1.001	[1.000, 1.003]
Openness	0.039	0.024	2.596	1	.107	1.040	[0.992, 1.091]
Neuroticism	0.027	0.020	1.853	1	.173	1.027	[0.988, 1.067]
Extraversion	0.040	0.018	5.299	1	.021	1.041	[1.006, 1.078]
Conscientiousness	0.003	0.020	0.025	1	.874	1.003	[0.964, 1.044]
Agreeableness	0.005	0.031	0.023	1	.880	1.005	[0.946, 1.067]
Age	-0.015	0.010	2.481	1	.115	0.985	[0.966, 1.004]
Gender (M = 1, F = 2)	-0.228	0.240	0.908	1	.341	0.796	[0.498, 1.273]
Education	0.094	0.105	0.806	1	.369	1.099	[0.895, 1.349]
Twitter use	0.356	0.126	7.997	1	.005	1.427	[1.115, 1.826]
Constant	-6.619	1.779	13.847	1	.000	0.001	

*N =* 671.

**Table 10:** Binary logistic regression: Predictors of whether participants had previously shared political stories they knew at the time were false, Study 2.

	*B*	*S*.*E*.	*Wald*	*df*	*p*	*Exp(B)*	*95% C*.*I*.*for EXP(B)*
New Media Literacy	0.009	0.011	0.733	1	.392	1.009	[0.988, 1.030]
Conservatism	0.001	0.001	1.255	1	.263	1.001	[0.999, 1.004]
Openness	0.030	0.036	0.667	1	.414	1.030	[0.959, 1.106]
Neuroticism	0.065	0.029	5.018	1	.025	1.067	[1.008, 1.129]
Extraversion	0.034	0.026	1.721	1	.190	1.034	[0.984, 1.087]
Conscientiousness	0.006	0.030	0.042	1	.837	1.006	[0.949, 1.067]
Agreeableness	-0.049	0.044	1.252	1	.263	0.952	[0.874, 1.037]
Age	-0.017	0.014	1.466	1	.226	0.983	[0.955, 1.011]
Gender (M = 1, F = 2)	-1.093	0.384	8.093	1	.004	0.335	[0.158, 0.712]
Education	-0.205	0.165	1.553	1	.213	0.815	[0.590, 1.125]
Twitter use	0.065	0.178	0.134	1	.714	1.067	[0.753, 1.514]
Constant	-4.125	2.583	2.551	1	.110	0.016	

*N =* 671.

### Discussion

For the main analysis, Study 2 replicates a number of key findings from Study 1. In particular, hypotheses 1, 2 and 4 were again unsupported by the results: consensus, authoritativeness, and new media literacy were not associated with self-rated likelihood of retweeting the disinformation stories. Evidence consistent with hypothesis 3 was again found, with higher levels of conservatism being associated with higher likelihood of retweeting. Again, the strongest predictor of likelihood of sharing was belief that the stories were true, while likelihood of having seen them before was again statistically significant. The only difference was in the role of personality: there was no association between Agreeableness (or any other personality variable) and likelihood of retweeting the material.

However, for self-reports of historical sharing of false political stories, the pattern of results was different. None of the previous results were replicated, and new predictors were observed for both un-knowing and deliberate sharing. For unintentional sharing, the link with higher levels of Twitter use makes sense, as higher usage confers more opportunities to accidentally share untruths. Higher Extraversion has also been found to correlate with higher levels of social media use [32] so the same logic may apply for that variable. For intentional sharing, the finding that men were more likely to share false political information is similar to findings from Study 1. The link with higher Neuroticism is less easy to explain: one possibility is that more neurotic people are more likely to share falsehoods that will reduce the chances of an event that they worry about (for example, spreading untruths about a political candidate who one is worried about being elected).

Given that these questions asked about past behaviour in general, and were not tied to the Twitter stimuli used in this study, it is not clear why the pattern of results should have differed from those in Study 1. One possibility is that the sample characteristics were different (this sample was younger, better educated, and drawn from a different source). Another realistic possibility, especially given the typically low effect sizes and large samples tested, is that these are simply ‘crud’ correlations [46] rather than useful findings. Going forward, it is likely to be more informative to focus on results that replicate across multiple studies or conceptually similar analyses.

## Study 3

Study 3 set out to repeat Study 1, but presented the materials as if they had been posted on Instagram rather than Facebook. Instagram presents an interesting contrast, as the mechanisms of engagement with material are different (for example there is no native sharing mechanism). Nonetheless, it has been identified as an important theater for disinformation operations [47]. Study 3 therefore sought to establish whether the same factors affecting sharing on Facebook also affect engagement with false material on Instagram. The study was completed online, using paid respondents sourced from the Prolific research panel.

### Method

The methodology exactly replicated that of Study 1, except in the case of details noted below. The planned analysis was revised to include the expanded set of predictors eventually used in Study 1 (see Table 4).

#### Materials

Measures and materials were the same as used in Study 1. The only difference from Study 1 was in the presentation of the three stimuli, which were portrayed as having been posted to Instagram rather than Facebook. For the consensus manipulation, ‘likes’ were used as the sole consensus indicator, and the appropriate icons for Instagram were used.

#### Procedure

The procedure replicated Study 1, save that in this case the NMLS was presented on a single page. Before participants saw each of the three disinformation items, the introductory paragraph stated “Imagine that you saw this post on your Instagram feed:” and they were asked to indicate the probability of them ‘liking’ the post.

#### Data screening and processing

Data submissions were initially obtained from 692 participants. A series of checks were performed to ensure data quality, resulting in a number of responses being excluded. Four individuals declined consent. Twenty-one were judged to have responded inauthentically, with the same scores to substantive sections of the questionnaire (‘straightlining’). Five did not indicate they were located in the UK. Ten were not active Instagram users: three individuals visited Instagram ‘not at all’ and seven ‘less often’ than every few weeks. Two participants responded unrealistically quickly, with response durations shorter than four minutes (the same value used as a speeding check by Qualtrics in Study 1). All of these respondents were removed, leaving *N* = 650. These checks and exclusions were carried out prior to any data analysis.

#### Participants

The target sample size was planned to exceed *N* = 614, as in Study 1. No attempt was made to recruit a demographically representative sample: instead, sampling quotas were used to ensure the sample was not homogenous with respect to education (pre-degree vs. undergraduate degree or above) and political preference (left, centre or right-wing orientation). Sampling was not stratified by age, given that Instagram use is associated with younger ages, and the number of older Instagram users in the Prolific pool was limited at the time the study was carried out. Additionally, participants had to be UK nationals resident in the UK; active Instagram users; and not participants in prior studies related to this one. Each participant received a reward of £1.25. Participant demographics are shown in Table 1 (column 3). For the focal analysis in this study, the sample size conferred 93.6% power to detect *R*^*2*^ = .04 in a multiple regression with 15 predictors (2-tailed, alpha = .05).

### Results

Descriptive statistics are summarised in Table 11. All scales had acceptable reliability. The main dependent variable, probability of liking, again had a very skewed distribution with a strong floor effect.

**Table 11:** Descriptive statistics for participant characteristics and response variables, Study 3.

					Range			
	*N*	*M*	*SD*	*α*	Potential	Actual	Skew	Kurtosis
Openness	650	24.27	5.71	.75	7–35	8–35	-0.29	-0.29
Conscientiousness	650	35.44	6.71	.84	10–50	15–50	-0.22	-0.24
Extraversion	650	26.66	7.31	.89	9–45	9–45	-0.06	-0.44
Agreeableness	649	27.09	4.29	.77	7–35	9–35	-0.71	0.78
Neuroticism	650	23.23	7.29	.88	8–40	8–40	0.13	-0.70
Conservatism	650	614.34	166.46	.79	0–1200	120–1130	-0.34	0.26
New Media Literacy	650	134.66	16.85	.93	35–175	69–174	-0.34	0.50
Probability of liking	650	5.68	4.47		3–33	3–29	2.27	5.94
Belief stories true	650	6.01	2.65		3–15	3–15	0.77	0.12
Likelihood seen before	650	5.19	2.85		3–15	3–15	1.36	1.20
Age	649	32.73	11.12			18–75	0.90	0.37

To simultaneously test hypotheses 1–4, a multiple regression analysis was carried out using the expanded predictor set from Study 1. Given inclusion of gender as a predictor variable, the three respondents who did not report their gender as either male or female were excluded from further analysis. The analysis, summarised in Table 12, indicated that the model explained 24% of the variance in self-reported likelihood of sharing the three disinformation items. Neither the authoritativeness of the story source, consensus information associated with the stories, nor consistency of the items with participant attitudes (conservatism) was a statistically significant predictor. Extraversion positively and Conscientiousness negatively predicted ratings of likelihood of sharing. In terms of demographic characteristics, men and younger participants reporting a higher likelihood of sharing. Finally, people reported a greater likelihood of sharing the items if they believed they were likely to be true, and if they thought they had seen them before.

**Table 12:** Predictors of rated likelihood of liking stimuli (extended predictor set), Study 3.

	*B*	*SE*		*β*	*t*	*p*
Constant	9.334	2.465			3.786	.000
New Media Literacy	-0.012	0.010		-0.046	-1.215	.225
Conservatism	0.001	0.001		0.044	1.016	.310
Openness	0.035	0.032		0.044	1.066	.287
Neuroticism	-0.050	0.028		-0.082	-1.795	.073
Extraversion	0.008	0.025		0.014	0.338	.736
Conscientiousness	-0.005	0.029		-0.008	-0.177	.860
Agreeableness	-0.093	0.041		-0.089	-2.282	.023
Authoritativeness	-0.517	0.315		-0.058	-1.641	.101
Consensus	-0.194	0.313		-0.022	-0.620	.535
Belief stories true	0.584	0.065		0.345	9.001	.000
Age	-0.047	0.016		-0.117	-3.024	.003
Gender (M = 1, F = 2)	-1.455	0.361		-0.155	-4.031	.000
Education	-0.081	0.151		-0.019	-0.537	.592
Instagram use	0.076	0.202		0.014	0.376	.707
Likelihood seen before	0.162	0.056		0.103	2.877	.004
*R*^*2*^			.241			
Adjusted *R*^*2*^			.223			
*F*			13.342			.000

Authoritativeness and Consensus conditions coded as dummy variables; higher = 1, lower = 0 in each case. *N =* 645.

Participants had also been asked about their historical sharing of untrue political stories, both unknowing and deliberate. Eighty five out of 650 (13.1%) participants who answered the question indicated that they had out ever ‘shared a political news story online that they later found out was made up’, while 50 out of 650 indicated they had shared one that they ‘thought AT THE TIME was made up’ (7.7%). Predictors of whether or not people had shared untrue material under both sets of circumstances were examined using logistic regressions, with the same sets of participant-level predictors.

Having unknowingly shared untrue material (Table 13) was significantly predicted by higher Extraversion, lower Conscientiousness and male gender. Having shared material known to be untrue at the time (Table 14) was significantly predicted by higher New Media Literacy, higher Conservatism, and higher Neuroticism.

**Table 13:** Binary logistic regression: Predictors of whether participants had previously shared political stories they subsequently discovered were false, Study 3.

	*B*	*S*.*E*.	*Wald*	*df*	*p*	*Exp(B)*	*95% C*.*I*.*for EXP(B)*
New Media Literacy	0.008	0.008	0.938	1	.333	1.008	[0.992, 1.023]
Conservatism	0.000	0.001	0.018	1	.893	1.000	[0.998, 1.002]
Openness	0.020	0.025	0.611	1	.434	1.020	[0.971, 1.071]
Neuroticism	0.036	0.022	2.722	1	.099	1.036	[0.993, 1.081]
Extraversion	0.057	0.019	9.404	1	.002	1.059	[1.021, 1.098]
Conscientiousness	-0.050	0.022	5.302	1	.021	0.951	[0.911, 0.993]
Agreeableness	0.004	0.030	0.018	1	.894	1.004	[0.947, 1.065]
Age	0.011	0.012	0.814	1	.367	1.011	[0.988, 1.034]
Gender (M = 1, F = 2)	-0.754	0.267	7.991	1	.005	0.471	[0.279, 0.794]
Education	-0.127	0.119	1.153	1	.283	0.880	[0.698, 1.111]
Instagram use	0.138	0.160	0.743	1	.389	1.148	[0.839, 1.570]
Constant	-3.402	1.856	3.360	1	.067	0.033	[0.992, 1.023]

*N* = 645.

**Table 14:** Binary logistic regression: Predictors of whether participants had previously shared political stories they knew at the time were false, Study 3.

	*B*	*S*.*E*.	*Wald*	*df*	*p*	*Exp(B)*	*95% C*.*I*.*for EXP(B)*
New Media Literacy	0.026	0.011	6.026	1	.014	1.026	[1.005, 1.048]
Conservatism	0.003	0.001	5.976	1	.015	1.003	[1.001, 1.005]
Openness	0.042	0.032	1.745	1	.187	1.043	[0.980, 1.111]
Neuroticism	0.075	0.028	7.372	1	.007	1.078	[1.021, 1.138]
Extraversion	0.042	0.023	3.344	1	.067	1.043	[0.997, 1.091]
Conscientiousness	-0.029	0.027	1.187	1	.276	0.971	[0.922, 1.023]
Agreeableness	-0.054	0.035	2.446	1	.118	0.947	[0.884, 1.014]
Age	-0.001	0.015	0.008	1	.927	0.999	[0.969, 1.029]
Gender (M = 1, F = 2)	-0.603	0.344	3.069	1	.080	0.547	[0.279, 1.074]
Education	-0.163	0.156	1.100	1	.294	0.849	[0.626, 1.152]
Instagram use	-0.191	0.191	0.997	1	.318	0.826	[0.568, 1.201]
Constant	-6.784	2.381	8.119	1	.004	0.001	

*N* = 645.

### Discussion

As in Studies 1 and 2, results were not consistent with hypotheses 1, 2 and 4: consensus, authoritativeness, and new media literacy were not associated with self-rated probability of liking the disinformation stories. In contrast to Studies 1 and 2, however, conservatism did not predict liking the stories. Belief that the stories were true was again the strongest predictor, while likelihood of having seen them before was again statistically significant. Among the personality variables, lower Agreeableness returned as a predictor of likely engagement with the stories, consistent with Study 1 but not Study 2. Lower age predicted likely engagement, a new finding, while being male predicted likely engagement as found in both in Study 1 and Study 2. Unlike Study 1 and Study 2, education had no effect.

With regard to historical accidental sharing, as in Study 3 higher Extraversion was a predictor, while as in Study 1 so was lower Conscientiousness. Men were more likely to have shared accidentally. Deliberate historical sharing was predicted by higher levels of New Media Literacy. This is counter-intuitive and undermines the argument that people share things because they know no better. In fact, in the context of deliberate deception, motivated individuals higher in digital literacy may actually be better equipped to spread untruths. Conservatism was also a predictor here. This could again be a reflection of the consistency hypothesis, given that there are high levels of conservative-oriented disinformation circulating. Finally, as in Study 3, higher Neuroticism predicted deliberate historical sharing.

## Study 4

Study 4 set out to repeat Study 1, but with a US sample and using US-centric materials. The purpose of this was to test whether the observed effects applied across different countries. The study was completed online, using as participants members of research panels sourced through the research company Qualtrics.

### Method

The methodology exactly replicated that of Study 1, except in the case of details noted below. The planned analysis was revised to include the expanded set of predictors eventually used in Study 1 (see Table 4).

#### Materials

Measures and materials were the same as used in Study 1. The only difference from Study 1 was in the contents of the three disinformation exemplars, which were designed to be relevant to a US rather than UK audience. Two of the stimuli were sourced from the website Infowars.com, while a third was a story described as untrue by the fact-checking website Politifact.com. In the same way as in Study 1, the right-wing focus of the stories was again established in pilot work where a US sample (*N* = 40) saw seven stories including these and rated their political orientation and likelihood of being shared. All were rated above the mid-point of an 11-point scale asking “To what extent do you think this post was designed to appeal to people with right wing (politically conservative) views?” anchored at “Very left wing oriented” and “Very right wing oriented”. For the least right-wing of the three stories selected, a one-sample *t*-test comparing the mean rating with the midpoint of the scale showed it was statistically significantly higher, *t*_(39)_ = 6.729, *p* < .001, *d* = 1.07). One of the stimuli, also used in Study 1–3, was titled “Revealed: UN plan to flood America with 600 million migrants”. One was titled “Flashback: Obama’s attack on internet freedom”, subtitled ‘Globalists, Deep State continually targeting America’s internet dominance’, featuring further anti- Obama, China and ‘Big Tech’ sentiment, and an image of Barack Obama apparently drinking wine with a person of East Asian appearance. The third was text based and featured material titled “Surgeon who exposed Clinton foundation corruption in Haiti found dead in apartment with stab wound to the chest”.

The materials used to manipulate authoritativeness (Facebook usernames shown as sources of the stories) were the same as used in Studies 1–3. These were retained because pilot work indicated that the higher and lower sets differed in authoritativeness for US audiences in the same way as for UK audiences. A sample of 30 US participants again each rated a selection of 9 usernames, including these 6, for the extent to which each was “likely to be an authoritative source—that is, likely to be a credible and reliable source of information”. A within-subjects *t*-test indicated that mean authoritativeness ratings for the ‘higher’ group were statistically significantly higher than the ‘lower’ group (*t*_(29)_ = -9.355 *p <* .001, *d*_*z*_ = 1.70).

#### Procedure

The procedure replicated Study 1, save that in this case the NMLS was presented across two pages.

#### Data screening and processing

Prior to delivery of the sample, Qualtrics performed a series of quality checks and ‘data scrubbing’ procedures to remove and replace participants with response patterns suggesting inauthentic or inattentive responding. These included speeding checks and examination of response patterns. On delivery of the initial sample (*N* = 660) further screening procedures were performed. Nine respondents were identified who had responded with the same scores to substantive sections of the questionnaire (‘straightlining’), and one who had not completed any of the personality items. Twelve respondents were not active Facebook users: Six reported using Facebook ‘not at all’ and a further six less often than ‘every few weeks’. All of these were removed, leaving *N* = 638. These checks and exclusions were carried out prior to any data analysis.

#### Participants

The target sample size was planned to exceed *N* = 614, as in Study 1. Qualtrics was contracted to provide a sample of active Facebook users that was broadly representative of the US population in terms of gender; education level; and age profile (18+). Sampling quotas were used to assemble a sample comprising approximately one third each self-describing as left-wing, centre and right-wing in their political orientation. Sampling errors on the part of Qualtrics led to over-recruitment of individuals aged 65 years, who make up 94 of the 160 individuals in the 60–69 age group. As a consequence, the 60–69 age group is itself over-represented in this sample compared to the broader US population. Participant demographics are shown in Table 1, column 4. For the focal analysis in this study, the sample size conferred 92.6% power to detect *R*^*2*^ = .04 in a multiple regression with 15 predictors (2-tailed, alpha = .05).

### Results

Descriptive statistics are summarised in Table 15. All scales had acceptable reliability. The main dependent variable, likelihood of sharing, again had a very skewed distribution with a strong floor effect.

**Table 15:** Descriptive statistics for participant characteristics and response variables, Study 4.

					Range			
	*N*	*M*	*SD*	*α*	Potential	Actual	Skew	Kurtosis
Openness	637	23.40	5.76	.74	7–35	9–35	0.05	-0.57
Conscientiousness	636	37.50	7.07	.81	10–50	15–50	-0.16	-0.67
Extraversion	635	27.51	6.84	.80	9–45	9–45	-0.15	0.14
Agreeableness	636	27.43	4.63	.71	7–35	10–35	-0.62	-0.24
Neuroticism	636	20.15	6.89	.82	8–40	8–40	0.30	-0.34
Conservatism	637	784.14	203.46	.83	0–1200	0–1200	-0.58	0.17
New Media Literacy	631	130.88	22.04	.95	35–175	40–171	-0.41	0.21
Likelihood of sharing	638	15.29	9.84		3–33	3–33	0.17	-1.25
Belief stories true	638	8.54	3.61		3–15	2–15	0.02	-1.00
Likelihood seen before	638	7.18	3.83		3–15	0–15	0.45	-1.02
Age	637	44.97	15.70			18–78	-0.02	-1.32

To simultaneously test hypotheses 1–4 a multiple regression analysis was carried out using the expanded predictor set from Study 1. Given inclusion of gender as a predictor variable, the one respondent who did not report their gender as either male or female was excluded from further analysis. The analysis, summarised in Table 16, indicated that the model explained 56% of the variance in self-reported likelihood of sharing the three disinformation items. Neither the authoritativeness of the story source, consensus information associated with the stories, nor consistency of the items with participant attitudes (conservatism) was a statistically significant predictor. Extraversion positively predicted ratings of likelihood of sharing. In terms of demographic characteristics, age was a significant predictor, with younger people reporting a higher likelihood of sharing. Finally, people reported a greater likelihood of sharing the items if they believed they were likely to be true, and if they thought they had seen them before.

**Table 16:** Predictors of rated likelihood of sharing stimuli (extended predictor set), Study 4.

	*B*	*SE*		*β*	*t*	*p*
Constant	-7.335	3.923			-1.87	.062
New Media Literacy	0.015	0.014		0.033	1.014	.311
Conservatism	0.001	0.002		0.03	0.837	.403
Openness	-0.009	0.062		-0.006	-0.152	.879
Neuroticism	0.061	0.054		0.043	1.135	.257
Extraversion	0.12	0.046		0.084	2.607	.009
Conscientiousness	0.069	0.056		0.05	1.222	.222
Agreeableness	-0.12	0.08		-0.057	-1.489	.137
Authoritativeness	-0.337	0.532		-0.017	-0.633	.527
Consensus	-0.148	0.536		-0.008	-0.276	.783
Belief stories true	1.329	0.101		0.487	13.163	.000
Age	-0.053	0.021		-0.084	-2.577	.010
Gender (M = 1, F = 2)	-0.689	0.574		-0.035	-1.2	.231
Education	0.243	0.229		0.031	1.057	.291
Facebook use	0.694	0.36		0.055	1.93	.054
Likelihood seen before	0.605	0.094		0.234	6.455	.000
*R*^*2*^			.560			
Adjusted *R*^*2*^			.549			
*F*			51.577			.000

Authoritativeness and Consensus conditions coded as dummy variables; higher = 1, lower = 0 in each case. *N =* 624.

Participants had also been asked about their historical sharing of untrue political stories, both unknowing and deliberate. Of the 638 participants, 185 (29.0%) indicated that they had ever ‘shared a political news story online that they later found out was made up’, while 132 out of 638 indicated they had shared one that they ‘thought AT THE TIME was made up’ (20.7%). Predictors of whether or not people had shared untrue material under both sets of circumstances were examined using logistic regressions, with the same sets of participant-level predictors.

Having unknowingly shared untrue material (Table 17) was significantly predicted by higher New Media Literacy, lower Conscientiousness, higher education, and higher levels of Facebook use. Having shared material known to be untrue at the time (Table 18) was significantly predicted by higher Extraversion, lower Agreeableness, younger age, and higher levels of Facebook use.

**Table 17:** Binary logistic regression: Predictors of whether participants had previously shared political stories they subsequently discovered were false, Study 4.

	*B*	*S*.*E*.	*Wald*	*df*	*p*	*Exp(B)*	*95% C*.*I*.*for EXP(B)*
New Media Literacy	0.015	0.005	7.936	1	.005	1.015	[1.005, 1.026]
Conservatism	0.000	0.001	0.466	1	.495	1.000	[0.999, 1.002]
Openness	0.012	0.022	0.275	1	.600	1.012	[0.969, 1.056]
Neuroticism	-0.005	0.020	0.072	1	.789	0.995	[0.956, 1.034]
Extraversion	0.020	0.017	1.366	1	.243	1.020	[0.987, 1.054]
Conscientiousness	-0.067	0.021	10.454	1	.001	0.935	[0.898, 0.974]
Agreeableness	-0.048	0.028	2.923	1	.087	0.953	[0.901, 1.007]
Age	-0.008	0.007	1.347	1	.246	0.992	[0.978, 1.006]
Gender (M = 1, F = 2)	0.068	0.207	0.108	1	.743	1.070	[0.713, 1.606]
Education	0.221	0.080	7.594	1	.006	1.247	[1.066, 1.459]
Facebook use	0.753	0.173	18.927	1	.000	2.124	[1.513, 2.982]
Constant	-4.115	1.495	7.577	1	.006	0.016	

*N =* 624.

**Table 18:** Binary logistic regression: Predictors of whether participants had previously shared political stories they knew at the time were false, Study 4.

	*B*	*S*.*E*.	*Wald*	*df*	*p*	*Exp(B)*	*95% C*.*I*.*for EXP(B)*
New Media Literacy	0.011	0.006	3.44	1	.064	1.011	[0.999, 1.023]
Conservatism	0.001	0.001	2.366	1	.124	1.001	[1.000, 1.003]
Openness	-0.024	0.026	0.855	1	.355	0.977	[0.929, 1.027]
Neuroticism	-0.008	0.024	0.102	1	.750	0.992	[0.947, 1.040]
Extraversion	0.045	0.020	4.88	1	.027	1.046	[1.005, 1.089]
Conscientiousness	-0.027	0.025	1.189	1	.276	0.973	[0.927, 1.022]
Agreeableness	-0.106	0.033	10.182	1	.001	0.900	[0.843, 0.960]
Age	-0.039	0.009	18.989	1	.000	0.962	[0.945, 0.979]
Gender (M = 1, F = 2)	-0.432	0.236	3.345	1	.067	0.649	[0.409, 1.031]
Education	0.148	0.088	2.853	1	.091	1.160	[0.976, 1.378]
Facebook use	0.344	0.173	3.972	1	.046	1.411	[1.006, 1.979]
Constant	-0.259	1.634	0.025	1	.874	0.772	

*N =* 624.

### Discussion

Again, the pattern of results emerging from Study 4 had some similarities but also some differences from Studies 1–3. Once again, hypotheses 1, 2 and 4 were unsupported by the results. Similarly to Study 3, but unlike Studies 1 and 2, conservatism (the proxy for consistency) did not predict sharing the stories. Belief that the stories were true, and likelihood of having seen them before, were the strongest predictors. Higher levels of Extraversion (a new finding) and lower ages (as in Study 3) were associated with higher reported likelihood of sharing the stimuli.

For historical sharing, for the first time–and counterintuitively–new media literacy was associated with higher likelihood of having shared false material unknowingly. As in Studies 1 and 2, lower Conscientiousness was also important. Counterintuitively, higher education levels were associated with higher unintentional sharing, as were higher levels of Facebook use. For intentional sharing, higher Extraversion was a predictor, as was lower Agreeableness, younger age and higher levels of Facebook use.

## General discussion

When interpreting the overall pattern of results from Studies 1–4, given the weakness of most of the associations, it is likely to be most useful to focus on relationships that are replicated across studies and disregard ‘one off’ findings. Tables 19–21 provide a summary of the statistically significant predictors in each of the studies. It is clear that two variables consistently predicted self-rated likelihood of sharing disinformation exemplars: belief that the stories were likely to be true, and likely prior familiarity with the stories. It is also clear that three key variables did not: markers of authority, markers of consensus and digital literacy.

**Table 19:** Predictors of self-reported likelihood of sharing, retweeting or liking stimuli across all studies.

	Study 1	Study 2	Study 3	Study 4
	(Facebook sharing)	(Twitter retweeting)	(Instagram liking)	(Facebook sharing)
New Media Literacy				
Conservatism	+	+		
Openness				
Neuroticism				
Extraversion				+
Conscientiousness				
Agreeableness	-		-	
Authoritativeness				
Consensus				
Belief stories true	+	+	+	+
Age			-	-
Gender (M = 1, F = 2)	-	-	-	
Education	-	-		
Platform use				
Likelihood seen before	+	+	+	+

+ denotes a statistically significant positive association,—a negative association.

**Table 20:** Predictors of un-knowingly sharing false political stories across all studies.

	Study 1	Study 2	Study 3	Study 4
	(Facebook sharing)	(Twitter retweeting)	(Instagram liking)	(Facebook sharing)
New Media Literacy				+
Conservatism				
Openness				
Neuroticism				
Extraversion		+	+	
Conscientiousness	-		-	-
Agreeableness	-			
Age	-			
Gender (M = 1, F = 2)			-	
Education				+
Platform use		+		+

+ denotes a statistically significant positive association,—a negative association.

**Table 21:** Predictors of knowingly sharing false political stories across all studies.

	Study 1	Study 2	Study 3	Study 4
	(Facebook sharing)	(Twitter retweeting)	(Instagram liking)	(Facebook sharing)
New Media Literacy			+	
Conservatism			+	
Openness				
Neuroticism		+	+	
Extraversion				+
Conscientiousness				
Agreeableness	-			-
Age	-			-
Gender (M = 1, F = 2)		-		
Education				
Platform use				+

+ denotes a statistically significant positive association,—a negative association.

Hypothesis 1 predicted that stories portrayed as coming from more authoritative sources were more likely to be shared. However, this was not observed in any of the four studies. One interpretation of this is that the manipulation failed. However, pilot work (see Study 1, Study 4) with comparable samples indicated that people did see the sources as differing in authoritativeness. The failure to find the predicted effect could also be due to use of simulated scenarios–though care was taken to ensure they resembled reality–or weaknesses in the methodology, such as the distributional properties of the dependent variables. However, consistent relationships between other predictors and the dependent variable were observed. Thus, the current studies provide no evidence that authoritativeness of a source influences sharing behaviour.

Hypothesis 2 predicted that stories portrayed as having a higher degree of consensus in audience reactions (i.e. high numbers of people had previously shared them) would be more likely to be shared. In fact, consensus markers had no effect on self-reported probability of sharing or liking the stories. Therefore, the current studies provide no evidence that indicators of ‘social proof’ influence participant reactions to the stimuli.

Hypothesis 3 was that people would be more likely to share materials consistent with their pre-existing beliefs. This was operationalised by measuring participants’ political orientation (overall level of conservatism) and using stimuli that were right-wing in their orientation. In Studies 1 and 2, more conservative people were more likely to share the materials. Further evidence for hypothesis 3 comes from the finding, across all studies, that level of belief the stories were “accurate and truthful” was the strongest predictor of likelihood of sharing. This is again in line with the consistency hypothesis: people are behaving in ways consistent with their beliefs. The finding from Study 3 that more conservative people were more likely to have historically shared material they knew to be untrue could also be in line with this hypothesis, given that a great many of the untrue political stories circulated online are conservative-oriented.

Hypothesis 4, that people lower in digital literacy would be more likely to engage with disinformation, was again not supported. As noted earlier, measurement of digital literacy is problematic. However, pilot work showed that the New Media Literacy Scale did differentiate between people with higher and lower levels of social media use in the expected manner, so it is likely to have a degree of validity. In Study 4, higher NMLS scores were associated with having unwittingly shared false material in the past, which is counterintuitive. However, this may be due to the fact that more digitally literate people should be more able to see that something was false in hindsight. Higher NMLS scores were also associated with deliberately sharing falsehoods in Study 3. This could be attributable to greater ease with which digitally literate individuals can do such things, if motivated to do so.

A number of other variables were included on an exploratory basis, or for the purpose of controlling for possible confounds. Of these, the most important was participants’ ratings of the likelihood that they had seen the stimuli before. This variable was originally included in the design so that any familiarity effects could be controlled for when evaluating the effect of other variables. In fact, rated likelihood of having seen the materials before was the second strongest predictor of likelihood of sharing it. It was a predictor in all four studies, and for the Facebook studies (1 and 4) it was the second most important variable. This is consistent with work on prior exposure to false material online, where prior exposure to fake news headlines increased participants’ ratings of their accuracy [44]. Furthermore, it has been found that prior exposure to fake-news headlines reduced participants’ ratings of how unethical it was to share or publish the material, even when it was clearly marked as false [43]. Thus, repeated exposure to false material may increase our likelihood of sharing it. It is known that repeated exposure to statements increases people’s subjective ratings of their truth [48]. However, there must be more going on here, because the regression analyses indicated that the familiarity effect was independent of the level of belief that it is true. When considering work that found that amplification of content by bot networks led to greater levels of human sharing [21], the implication is that repeated actual exposure to the materials is what prompts people to share it, not metrics of consensus such as the number of likes or shares displayed beside an article.

Of the five dimensions of personality measured, four (Agreeableness, Extraversion, Neuroticism and Conscientiousness were predictors of either current or historical sharing in one or more studies. Consistent with findings from [24], Studies 1 and 3 found that lower Agreeableness was associated with greater probability of sharing or liking the stories. It was also associated with accidental historical sharing in Study 1, and deliberate historical sharing in Studies 1 and 4. In contrast to this, past research on personality and social media behaviour indicates that *more* agreeable people are more likely to share information on social media: [49] reported that its role in this was mediated by trust, while [32] found that higher Agreeableness was associated with higher levels of social media use in general. Given those findings, it is likely that the current results are specific to disinformation stimuli rather than social sharing in general. Agreeableness could potentially interact with the source of the information: more agreeable people might conceivably be more eager to please those close to them, However, while it is possible that Agreeableness interacted in some way with the framing of the material having been shared by ‘a friend’ in Study 1, Study 3 had no such framing. More broadly, the nature of the stories may be important: disinformation items are normally critical or hostile in their nature. This may mean they are more likely to be shared by disagreeable people, who themselves may be critical in their outlook and not concerned about offending others. Furthermore, Agreeableness is associated with general trusting behaviour. It may be that disagreeable people are therefore more likely to endorse conspiracist material, or other items consistent with a lack of trust in politicians or other public figures.

Lower Conscientiousness was associated with accidental historical sharing of false political stories in Studies 1, 3 and 4. This is unsurprising, as less conscientious people would be less likely to check the veracity of a story before sharing it. The lack of an association with deliberate historical sharing reinforces this view.

Higher Extraversion was associated with probability of sharing in Study 4, with accidental historical sharing in Study 2 and 3, and with deliberate historical sharing in Study 4. Higher Neuroticism was associated with historical deliberate sharing in Studies 2 and 3. All these relationships may simply reflect a higher tendency on the part of extraverted and neurotic individuals to use social media more [32].

There are clearly some links between personality and sharing of disinformation. However, the relationships are weak and inconsistent across studies. It is possible that different traits affect different behaviours: for example low Conscientiousness is associated with accidental but not deliberate sharing, while high Neuroticism is associated with deliberate but not accidental sharing. Thus, links between some personality traits and the spread of disinformation may be context- and motivation- specific, rather than reflecting blanket associations. However, lower Agreeableness–and to a lesser extent higher Extraversion–may predict an overall tendency to spread this kind of material.

Demographic variables were also measured and included in the analyses. Younger individuals rated themselves as more likely to engage with the disinformation stimuli in Studies 3 and 4, and were more likely to have shared untrue political stories in the past either accidentally (Study 1) or deliberately (Studies 1 and 4). This runs counter to findings that older adults were much more likely to have spread material from ‘fake news’ domains [16]. It is possible that the current findings simply reflect a tendency of younger people to be more active on social media.

People with lower levels of education reported a greater likelihood of sharing the disinformation stories in Studies 1 and 2. Counterintuitively, more educated people were more likely to have accidentally shared false material in the past (Study 4). One possible explanation is that more educated people are more likely to have realised that they had done this, so the effect in Study 4 reflects an influence on reporting of the behaviour rather than on the behaviour itself.

In each of Studies 1, 2 and 3, men reported a greater likelihood of sharing or liking the stimuli. Men were also more likely to have shared false material in the past unintentionally (Study 3) or deliberately (Study 2). Given its replicability, this would seem to be a genuine relationship, but one which is not easy to explain.

Finally, the level of use of particular platforms (Facebook, Twitter or Instagram) did not predict likelihood of sharing the stimuli in any study. Level of use of Twitter (Study 2) predicted accidental sharing of falsehoods, while Facebook use predicted both accidental and deliberate sharing (Study 4). For historical sharing, this may be attributable to a volume effect: the more you use the platforms, the more likely you are to do these things. It should be noted that the level of use metric lacked granularity and had a strong ceiling effect, with most people reporting the highest use level in each case.

In all four studies, a minority of respondents indicated that they had previously shared political disinformation they had encountered online, either by mistake or deliberately. The proportion who had done each varied across the four studies, likely as a function of the population sampled (13.1%-29.0% accidentally; 6.2%-20.7% deliberately), but the figures are a similar magnitude to those reported elsewhere [31,16]. Even if the proportion of social media users who deliberately share false information is just 6.2%, the lowest figure found here, then that is still a very large number of people who are actively and knowingly spreading untruths.

The current results indicate that a number of variables predict onward sharing of disinformation. However, most of these relationships are very small. It has been argued that the minimum effect size for a predictor that would have real-world importance in social science data is *β* = .2 [42]. Considering the effect sizes for the predictors in Tables 4, 8, 12 and 17, only belief that the stories are true exceeds this benchmark in every study, while probability of having seen the stories before exceeded it in Studies 1 and 4. None of the other relationships reported exceeded the threshold. This has implications for the practical importance of these findings, in terms of informing interventions to counteract disinformation.

### Practical implications

Some of the key conclusions in this set of studies arise from the failure to find evidence supporting an effect. Proceeding from such findings to a firm conclusion is a logically dangerous endeavour: absence of evidence is not, of course, evidence of absence. However, given the evidence from pilot studies that the manipulations were appropriate; the associations of the dependent measures with other variables; and the high levels of power to detect the specified effects, it is possible to say with some confidence that hypotheses 1, 2 and 4 are not supported by the current data. This means that the current project does not provide any evidence that interventions based on these would be of value.

This is particularly important for the findings around digital literacy. Raising digital media literacy is a common and appealing policy position for bodies concerned with disinformation (e.g. [1]). There is evidence from a number of trials that it can be effective in the populations studied. However, no support was found here for the idea that digital literacy has a role to play in the spread of disinformation. This could potentially be attributed to the methodology in this study. However, some participants– 288 in total across all four studies–reported sharing false political stories that they knew at the time were made up. It is hard to see how raising digital literacy would reduce such deliberate deception. Trying to raise digital literacy across the population is therefore unlikely to ever be a complete solution.

There is evidence that consistency with pre-existing beliefs can be an important factor, especially in relation to beliefs that disinformation stories are accurate and truthful. This implies that interventions are likely to be most effective when targeted at individuals who already hold an opinion or belief, rather than trying to change people’s minds. While this would be more useful to those seeking to spread disinformation, it could also give insights into populations worth targeting with countermessages. Targeting on other variables–personality or demographic–is unlikely to be of value given the low effect sizes. While these variables (perhaps gender and Agreeableness in particular) most likely do play a role, their relative importance seems so low that the information is unlikely to be useful in practice.

Alongside other recent work [43,44], the current findings suggest that repeated exposure to disinformation materials may increase our likelihood of sharing it, even if we don’t believe it. The practical implication would be that to get a message repeated online, one should repeat it many times (there is a clear parallel with the ‘repeat the lie often enough’ maxim regarding propaganda). Social proof (markers of consensus) seems unimportant based on current findings, so there is no point in trying to manipulate the numbers next to a post as sometimes done in online marketing. What might be more effective is to have the message posted many times (e.g. by bots) so that people had a greater chance of coming across it repeatedly. This would be true both for disinformation and counter-messages.

### Limitations

As a scenario-based study, the current work has a number of limitations. While it is ethically preferable to field experiments, it suffers from reduced ecological validity and reliance on self-reports rather than genuine behaviour. Questions could be asked, for example, about whether the authoritativeness and consensus manipulations were sufficiently salient to participants (even though they closely mirrored the presentation of this information in real-life settings). Beyond this, questions might be raised about the use of self-reported likelihood of sharing: does sharing intention reflect real sharing behaviour? In fact, there is evidence to suggest that it does, with recent work finding that self-reported willingness to share news headlines on social media paralleled the actual level of sharing of those materials on Twitter [50].

The scenarios presented were all selected to be right-wing in their orientation, whereas participants spanned the full range from left to right in their political attitudes. This means that consistency was only evaluated with respect to one pole of the right-left dimension. There are a number of other dimensions that have been used as wedge issues in real-world information operations: for example, support for the Black Lives Matter movement; climate change; or for or against Britain leaving the European Union. The current research only evaluated consistency between attitudes and a single issue. A better test of the consistency hypothesis would be to extend that to evaluation of consistency between attitudes and some of those other issues.

A key issue is the distributions of the main outcome variables, which were heavily skewed with strong floor effects. While they still had sufficient sensitivity to make the regression analyses meaningful, they also meant that any effects found were likely to be attenuated. It may thus be that the current findings underestimate the strength of some of the associations reported.

Another measurement issue is around the index of social media use (Facebook, Twitter, Instagram). As Table 1 shows, in three of the studies over 60% of respondents fall into the highest use category. Again, this weakens the sensitivity of evaluations of these variables as predictors of sharing disinformation.

In order to identify variables associated with sharing disinformation, this research programme took the approach of presenting individuals with examples of disinformation, then testing which of the measured variables was associated with self-reported likelihood of sharing. A shortcoming of this approach is that it does not permit us to evaluate whether the same variables are associated with sharing true information. An alternative design would be to show participants either true or false information, and examine whether the same constructs predict sharing both. This would enable identification of variables differentially impacting the sharing of disinformation but not true information. Complexity arises, however, from the fact that whether a story can be considered disinformation, misinformation, or true information, depends on the observer’s perspective. False material deliberately placed online would be categorized as disinformation. A social media user sharing it in full knowledge that it was untrue would be sharing disinformation. However, if they shared it believing it was actually true, then from an observer’s perspective this would be technically categorised as misinformation (defined as “the inadvertent sharing of false information” [1, p.10]). In fact, from the user’s perspective, it would be true information (because they believe it) even though an omniscient observer would know it was actually false. This points to the importance of further research into user motivations for sharing, which are likely to differ depending on whether or not they believe the material is true.

In three of the four studies (Studies 1,2, 4), the stimulus material was introduced as having been posted by a friend who wanted them to share it. This is likely to have boosted the rates of self-reported likelihood of sharing in those studies. Previous work has shown that people rate themselves as more likely to engage with potential disinformation stories posted by a friend, as opposed to a more distant acquaintance [24]. To be clear, this does not compromise the testing of hypotheses in those studies (given that the framing was the same for all participants, in all conditions). It is also a realistic representation of how we may encounter material like this in our social media feeds. However, it does introduce an additional difference between Studies 1, 2 and 4 when compared with Study 3. It would be desirable for further work to check whether the same effects were found when messages were framed as having been posted by people other than friends.

Finally, the time spent reading and reacting to the disinformation stimuli was not measured. It is possible that faster response times would be indicative of more use of heuristics rather than considered thought about the issues. This could profitably be examined, potentially in observational or simulation studies rather than using self-report methodology.

### Future work

A number of priorities for future research arise from the current work. First, it is desirable to confirm these findings using real-world behavioural measures rather than simulations. While it is not ethically acceptable to run experimental studies posting false information on social media, it would be possible to do real-world observational work. For example, one could measure digital literacy in a sample of respondents, then do analyses of their past social media sharing behaviour.

Another priority revolves around those individuals who knowingly share false information. Why do they do this? Without understanding the motivations of this group, any interventions aimed at reducing the behaviour are unlikely to be successful. As well as being of academic interest, motivation for sharing false material has been flagged as a gap in our knowledge by key stakeholders [7].

The current work found that men were more likely to spread disinformation than women. At present, it is not clear why this was the case. Are there gender-linked individual differences that influence the behaviour? Could it be that the subject matter of disinformation stories is stereotypically more interesting to men, or that men think their social networks are more likely to be interested in or sympathetic to them?

While the focus in this paper has been on factors influencing the spread of untruths, it should be remembered that ‘fake news’ is only one element in online information operations. Other tactics and phenomena, such as selective or out-of-context presentation of true information, political memes, and deliberately polarising hyperpartisan communication, are also prevalent. Work is required to establish whether the findings of this project related to disinformation, also apply to those other forms of computational propaganda. Related to this, it would be of value to establish whether the factors found here to influence sharing of untrue information, also influence the sharing of true information. This would indicate whether there is anything different about disinformation, and also point to factors that might influence sharing of true information that is selectively presented in information operations.

### Conclusion

The current work allows some conclusions to be drawn about the kind of people who are likely to further spread disinformation material they encounter on social media. Typically, these will be people who think the material is likely to be true, or have beliefs consistent with it. They are likely to have previous familiarity with the materials. They are likely to be younger, male, and less educated. With respect to personality, it is possible that they will tend to be lower in Agreeableness and Conscientiousness, and higher in Extraversion and Neuroticism. With the exception of consistency and prior exposure, all of these effects are weak and may be inconsistent across different populations, platforms, and behaviours (deliberate v. innocuous sharing). The current findings do not suggest they are likely to be influenced by the source of the material they encounter, or indicators of how many other people have previously engaged with it. No evidence was found that level of literacy regarding new digital media makes much difference to their behaviour. These findings have implications for how governments and other bodies should go about tackling the problem of disinformation in social media.
